# Modern Advances in Microendodontics: The State of the Art

**DOI:** 10.3390/bioengineering10070789

**Published:** 2023-07-01

**Authors:** Alfredo Iandolo

**Affiliations:** Department of Medicine, Surgery and Dentistry, University of Salerno, 84084 Salerno, Italy; aiandolo@unisa.it

The concept of “minimally invasive” advances is becoming increasingly popular in the endodontic field. This concept comprises all endodontic treatment stages from diagnosis to root canal filling [1,2,3].

The success of endodontics depends on all phases. Only when every single step is performed perfectly can long-term success be achieved [4,5].

After diagnosis, with the help of modern radiological technologies, such as 3D CBCT and image processing software, it is possible to view every detail of the teeth we must treat.

Moreover, knowing about the problems in advance will allow the clinician to provide a proper treatment plan, even in extreme cases. In this way, the clinician may learn that the tooth cannot be saved and, therefore, must be extracted [6].

Unfortunately, with traditional 2D radiology, difficult and unusual cases cannot be visualised properly. Hence, an accurate treatment plan will be unachievable.

Once the diagnostic phase has been completed, the access cavity phase follows.

For several years, attempts have been made to conservatively carry this out to avoid weakening the residual tooth’s structure. Knowing about endodontic anatomy is important for performing the modern access cavity technique, and it is essential to take advantage of modern technologies, such as operating microscopes, powerful lights, and ultrasound systems with specific ultrasonic tips [7,8,9,10].

Thus, it is possible to achieve success by eliminating iatrogenic errors.

Next, shaping is performed, which involves minimally invasive “conservative shaping”. The latest generation of rotating files are used for this, which are much more flexible, thereby reducing the fracture risk [11,12,13,14].

Modern endodontics aims to eliminate damaged tissue and bacteria from the endodontic space. This goal can be achieved when conservative shaping is combined with powerful active irrigation.

After shaping, the 3D cleaning phase begins. In this phase, irrigants remove the smear layer, vital or necrotic tissues and bacteria in a single or biofilm form. Irrigants alone are not effective at reaching complex areas of the endodontic space. Instead, by employing powerful activation, irrigants can reach even in the most microscopic spaces. Different activation techniques exist, including heat, ultrasound, sonic, subsonic, laser systems, etc. [15,16,17,18,19,20,21,22].

The final stage of endodontic treatment includes filling the spaces that have been shaped and cleaned. Recently, new sealers have been introduced for the obturation phase, such as biosealers, which have unique characteristics.

During the filling phase, it is important to use techniques that seal the entire endodontic anatomy as much as possible, even microscopic ones such as the dentinal tubules [23,24,25,26,27,28].

In conclusion, if they are employed well, modern technologies, materials and recent clinical protocols ensure the success of endodontic treatments.
